# Baseline characteristics of patients with SLE with and without lupus nephritis from the Latin American multiethnic GLADEL 2.0 cohort

**DOI:** 10.1136/lupus-2025-001878

**Published:** 2026-04-20

**Authors:** Guillermo J Pons-Estel, Romina E Nieto, Rosana Quintana, Rosa Serrano Morales, Eduardo F Borba, Lucia Hernández, Guillermina B Harvey, Karen Roberts, Diana C Fernández-Ávila, Marina Scolnik, Carmen Funes Soaje, Cintia Otaduy, Verónica Saurit, Valeria Arturi, Guillermo A Berbotto, Luciana González Lucero, Eduardo Kerzberg, Graciela N Gómez, Cecilia N Pisoni, Vicente Juárez, Joaquín Martínez Serventi, Carolina Ayelen Isnardi, Luciana Parente Costa Seguro, Ana Carolina O S Montadon, Odirlei André Monticielo, Henrique A Mariz, Francinne Machado Ribeiro, Edgard Torres dos Reis-Neto, Iris Guerra, Loreto Massardo, Gustavo Aroca Martínez, Antonio Iglesias Gamarra, Carlos A Cañas, Gerardo Quintana López, Carlos E Toro Gutiérrez, Mario J Moreno Álvarez, Miguel A Saavedra, Margarita Portela Hernández, Mario Pérez Cristóbal, Hilda Fragoso Loyo, Yaneli Juárez-Vicuña, Yelitza C González-Bello, Carlos Abud-Mendoza, Jorge A Esquivel-Valerio, Patricia Langjahr, Astrid Paats, Claudia Mora Trujillo, Manuel F Ugarte-Gil, Armando Calvo-Quiroz, Roberto Muñoz Louis, Martin Rebella, Álvaro Danza, Urbano Sbarigia, Federico Zazzetti, Ashley Orillion, José A Gómez Puerta, Graciela S Alarcon, Bernardo A Pons-Estel

**Affiliations:** 1 Grupo Oroño - Centro Regional de Enfermedades Autoinmunes y Reumáticas (GO-CREAR), Rosario, Argentina; 2 Rheumatology Division, Hospital das Clinicas, Faculdade de Medicina da Universidade de São Paulo (HCFMUSP), São Paulo, Brazil; 3 Departamento de Psicología y Sociología, Universidad de Zaragoza, Zaragoza, Spain; 4 Instituto de Investigaciones Teóricas y Aplicadas, Facultad de Ciencias Económicasy Estadística, Universidad Nacional de Rosario, Rosario, Argentina; 5 Instituto enfermedades inflamatorias e inmunomediadas - Hospital Universitario Mayor Méderi - Universidad del Rosario, Bogotá, Colombia; 6 Sección Reumatología, Hospital Italiano de Buenos Aires, Buenos Aires, Argentina; 7 Hospital Italiano de Córdoba, Córdoba, Argentina; 8 Servicio de Reumatología Hospital Córdoba y Sanatorio Allende, Facultad de Ciencias Médicas, Universidad Nacional de Córdoba, Córdoba, Argentina; 9 Hospital Privado Universitario de Córdoba, Córdoba, Argentina; 10 Hospital San Martin de La Plata, Buenos Aires, Argentina; 11 Sanatorio Británico, Rosario, Argentina; 12 Hospital Padilla, San Miguel de Tucumán, Tucumán, Argentina; 13 Hospital General de Agudos Doctor José María Ramos Mejía, Buenos Aires, Argentina; 14 Instituto de Investigaciones Médicas Dr. Alfredo Lanari, Universidad de Buenos Aires, Buenos Aires, Argentina; 15 Centro de Educación Médica e Investigaciones Clínicas (CEMIC) ‘‘Norberto Quirno’’, Buenos Aires, Argentina; 16 Servicio de Reumatología, Hospital Señor del Milagro, Salta, Argentina; 17 Hospital General de Agudos Dr. Juan A. Fernández, Buenos Aires, Argentina; 18 Centro Traumatológico Bariloche, Bariloche, Rio Negro, Argentina; 19 Serviço de Reumatologia, Hospital das Clínicas da Universidade Federal de Goias, Goiânia, Brazil; 20 Serviço de Reumatologia, Hospital de Clínicas de Porto Alegre, Universidade Federal do Rio Grande do Sul, Porto Alegre, Brazil; 21 Universidade Federal de Pernambuco, Recife, Brazil; 22 Hospital Universitario Pedro Ernesto, Universidade do Estado do Rio de Janeiro, Rio de Janeiro, Brazil; 23 Rheumatology Divison, Department of Medicine, Escola Paulista de Medicina / Universidade Federal de São Paulo, São Paulo, Brazil; 24 Hospital del Salvador, Santiago, Chile; 25 Hospital Dr Franco Ravera Zunino, Rancagua, Chile; 26 Facultad de Medicina y Ciencias, Universidad San Sebastián, Santiago, Chile; 27 Clínica de la Costa y Universidad Simón Bolivar. Universidad Simón Bolívar Facultad de Ciencias de la Salud, Barranquilla, Colombia; 28 Fundación Valle del Lili, Universidad Icesi, Cali, Colombia; 29 Facultad de Medicina, Universidad Nacional de Colombia, Servicio de Reumatología, Hospital Universitario Fundación Santa Fe de Bogotá, Hospital Universitario Nacional de Colombia, Bogotá, Colombia; 30 Centro de Referencia en Osteoporosis & Reumatología, Pontificia Universidad Javeriana Cali, Cali, Colombia; 31 Division of Rheumatology, McMaster University, St. Joseph's Healthcare Hamilton, Hamilton, Ontario, Canada; 32 Universidad de Especialidades Espíritu Santo, Guayaquil, Ecuador; 33 División de Investigación en Salud, Hospital de Especialidades Dr. Antonio Fraga Mouret, Centro Médico Nacional La Raza, Instituto Mexicano del Seguro Social, Ciudad, México; 34 Hospital de Especialidades del Centro Médico Nacional Siglo XXI, Instituto Mexicano del Seguro Social (IMSS), Ciudad, México; 35 Departamento de Inmunología y Reumatología, Instituto Nacional de Ciencias Médicas y Nutrición Salvador Zubirán, Ciudad, México; 36 Instituto Nacional de Cardiología Ignacio Chávez, Departamento de Inmunología, Ciudad, México; 37 Centro de Estudios de Investigación Básica y Clínica, S.C., Guadalajara, México; 38 Facultad de Medicina de la Universidad Autónoma de San Luis Potosí y Hospital Central Dr. Ignacio Morones Prieto, San Luis Potosí, México; 39 Internal Medicine Department and Rheumatology Service, Hospital Universitario Dr. José Eleuterio González, Universidad Autónoma de Nuevo León, Monterrey, México; 40 Facultad de Ciencias Médicas, Universidad Nacional de Asunción, San Lorenzo, Asunción, Paraguay; 41 Servicio de Reumatología, Hospital Nacional Edgardo Rebagliati Martins (EsSalud), Lima, Peru; 42 Rheumatology Department, Hospital Guillermo Almenara Irigoyen, EsSalud, Lima, Peru; 43 Grupo Peruano de Estudio de Enfermedades Autoinmunes Sistémicas, Universidad Científica del Sur, Lima, Peru; 44 Servicio de Inmunología y Reumatología, Hospital Nacional Cayetano Heredia, Universidad Peruana Cayetano Heredia, Lima, Peru; 45 Hospital Docente Padre Billini, Santo Domingo, Dominican Republic; 46 Unidad de Enfermedades Autoinmunes Sistémicas, Hospital de Clínicas, Facultad de Medicina, Universidad de la República, Montevideo, Uruguay; 47 Médica Uruguaya, Facultad de Medicina, Universidad de la República, Montevideo, Uruguay; 48 Johnson & Johnson, Brussels, Belgium; 49 Johnson & Johnson, Horsham, Pennsylvania, USA; 50 Johnson & Johnson, Spring House, Pennsylvania, USA; 51 Departamento de Reumatología, Hospital Clinic, Barcelona, Spain; 52 Division of Clinical Immunology and Rheumatology, Department of Medicine, The University of Alabama at Birmingham Marnix E. Heersink School of Medicine, Birmingham, Alabama, USA; 53 Departamento de Medicina, Facultad de Medicina, Universidad Peruana Cayetano Heredia, Lima, Peru

**Keywords:** Lupus Nephritis, Lupus Erythematosus, Systemic, Risk Factors

## Abstract

**Objective:**

To describe baseline characteristics of a multinational, multiethnic Latin American cohort of SLE patients with and without lupus nephritis (LN) from Latin American Group for the Study of Lupus (GLADEL) 2.0.

**Methods:**

GLADEL 2.0 is an observational prevalent and incident cohort. Adult patients (≥18 years) with SLE who fulfilled the 1982/1997 American College of Rheumatology (ACR) and/or the 2012 Systemic Lupus International Collaborating Clinics (SLICC) classification criteria from 43 centres in 10 Latin American countries were categorised into four groups: (1) without LN, (2) prevalent inactive LN, (3) prevalent active LN and (4) incident LN. Baseline demographics, clinical manifestations, treatments, disease activity and damage were recorded. A cross-sectional analysis and logistic regression was conducted to identify factors associated with LN and cumulative damage (SLICC/ACR Damage Index (SDI)).

**Results:**

Among 1083 patients, 89.6% were female, median age at diagnosis was 27 years, median disease duration was 66.2 months and 65.0% were Mestizo. Patients with LN (n=653) were younger and had shorter disease duration than patients without LN. LN was negatively associated with older age at diagnosis (OR, 0.98) and discoid rash (OR, 0.53) and positively associated with male gender (OR, 1.91), Mestizo ethnicity (OR, 1.46), comorbidities (OR, 3.2), thrombocytopenia (OR, 3.44), anti–double-stranded DNA (anti-dsDNA) positivity (OR, 3.23) and low complement (OR, 1.54). SDI ≥1 was associated with older age at diagnosis, longer disease duration, cumulative prednisone dose and cyclophosphamide use.

**Conclusions:**

Male sex, Mestizo ethnicity, comorbidities, thrombocytopenia, anti-dsDNA positivity and low complement levels were associated with LN occurrence. Cumulative damage was associated with older age at diagnosis, longer disease duration, higher cumulative dose of prednisone and the use of cyclophosphamide.

**Trial registration number:**

NCT04534647.

**WHAT IS ALREADY KNOWN ON THIS TOPIC**

**WHAT THIS STUDY ADDS**

- Patients with comorbidities, thrombocytopenia and positive anti-double-stranded DNA (anti-dsDNA) antibodies were three times more likely to have lupus nephritis (LN). Males were almost twice as likely as females to have LN, as were Mestizos compared with Caucasians. Each additional year of age at diagnosis was associated with a 2% decrease in the probability of LN, indicating that younger patients are at increased risk. The presence of a discoid rash was associated with an almost one-half reduction in the probability of LN, suggesting that it may be a marker of a milder phenotype of the disease.

**HOW THIS STUDY MIGHT AFFECT RESEARCH, PRACTICE OR POLICY**

- This study alerts clinicians caring for these ethnic groups to the relevance of demographic and clinical characteristics in the development of renal involvement.

## Introduction

 SLE is an autoimmune disease characterised by a broad spectrum of clinical manifestations, varying from mild to major organ involvement, which negatively impacts patients’ health-related quality of life and their survival. In Latin America (LATAM), SLE exhibits particular characteristics, including a higher prevalence, earlier onset and more aggressive course, compared with patients from developed countries.^1^ These differences are influenced by multiple determinants, such as the genetic susceptibility of Mestizo populations, exposure to specific environmental factors and, most notably, inequities in accessing medical care.^2 3^

The Latin American Group for the Study of Lupus (GLADEL, for *Grupo Latino Americano de Estudio del Lupus*) has previously described these data in another cohort of patients with SLE from the region. The influence of ethnicity, familial aggregation, gender, childhood and late-onset disease has been described, as well as the benefits of antimalarials in the prevention of flares, a decrement in disease activity, protection against severe infections and better survival.^14^,^17^ Lupus nephritis (LN) outcomes are influenced by several factors, including race and ethnicity, access to healthcare and environmental factors.^1 18^

LATAM is a large subcontinent with significant ethnic admixtures between and within countries and with diverse socioeconomic, educational and demographic realities that influence the severity of SLE. Population-based cohorts provide a rationale for recognising the different characteristics of the disease and for establishing guidelines for SLE treatment. However, not all data are generalisable, demonstrating the need to establish regional SLE cohorts to better understand disease features, address health inequities within these populations and provide a community-based approach to SLE management. For these reasons, we have now assembled a large, multinational, multiethnic cohort of patients with SLE with and without LN (GLADEL 2.0). This cohort represents 10 LATAM countries^18^ and it builds on the legacy of the original GLADEL cohort, but with enhanced methodology and updated data to reinforce and expand on previous observations regarding ethnicity-related differences in disease manifestations, while introducing novel elements such as a centralised biomarker collection. The primary objective of this communication is to describe the baseline sociodemographic, clinical and therapeutic characteristics of this cohort. Secondary objectives are to evaluate the factors associated with the occurrence of LN and with damage accrual at cohort entry.

## Methods

### Centres selection

A large, multinational, multiethnic, LATAM cohort of patients with SLE with and without LN (GLADEL 2.0) was constituted.^18^ Reference centres in rheumatology, internal medicine and nephrology with expertise in SLE, academic profiles and training programmes were included. The 43 participating centres are distributed in 10 LATAM countries: Argentina, Brazil, Chile, Colombia, Dominican Republic, Ecuador, Mexico, Paraguay, Peru and Uruguay, as shown in online supplemental figure 1.

### Patient selection

Between May 2019 and October 2023, patients ≥18 years of age, with a diagnosis of SLE according to either the 1982/1997 American College of Rheumatology (ACR)^19 20^ and/or the 2012 Systemic Lupus International Collaborating Clinics (SLICC)^21^ classification criteria, who agreed to participate and signed the corresponding informed consent, were included in the study. Annual follow-up is being done in these patients. Patients were categorised into four subsets according to the presence of LN: *Group I:* patients with SLE, *without renal involvement* (never had persistent proteinuria, cellular casts or impaired renal function); *Group II*: patients with SLE, with *prevalent LN* (at any time during their disease course), *currently inactive* (referred to patients with a prior LN diagnosis who had fully resolved these criteria at enrolment); *Group III*: patients with SLE, with *prevalent LN* (at any time during their disease course), *currently active* (defined as persistent proteinuria >0.5 g/day or >3+ on dipstick if quantification was unavailable and/or presence of cellular casts at cohort entry) and *Group IV*: patients with SLE, with *incident LN* (maximum duration, 3 months, untreated with immunosuppressants and with a mandatory renal biopsy); immunosuppressive treatment was initiated according to clinical indication and not delayed by study procedures. Information on prior renal involvement was obtained from medical record review at each site, with verification by site investigators and centralised data control committee to minimise misclassifications. Patients with concomitant Sjögren’s disease and antiphospholipid syndrome were included. Patients with other systemic autoimmune diseases or overlapping syndromes (rheumatoid arthritis, systemic sclerosis, dermato-polymyositis, systemic vasculitis and others), who were pregnant at the time of inclusion or had an active urinary or a systemic infection (including hepatitis B and C, HIV or active COVID-19 infection) were excluded.

### Database

Data were collected using ARTHROS-Web (www.arthrosoft.com), a modified version of the ARTHROS software. This is a validated and user-friendly database developed by Argentine rheumatologists. The identity of the patients is confidential, and the exported data are anonymised.

### Clinical information

At baseline, sociodemographic factors (sex, ethnicity, socioeconomic level assessed by Graffar method,^22^ educational level, occupation), physical examination and lifestyle habits, cumulative lupus criteria, clinical manifestations (general, skin and mucosal, musculoskeletal, renal, renal histopathologic findings, neuropsychiatric, ocular, serositis, cardiovascular, pulmonary, gastrointestinal, haematologic and maternal-fetal, infections, hospitalisations), comorbidities (arterial hypertension, diabetes mellitus, dyslipidaemia), treatment regimens (specific for lupus manifestations and treatments for other conditions) and traditional serological and urinary biomarkers performed locally (haematological, chemistry, urinalysis, autoantibodies, complement) were recorded. The clinical data examined correspond to cumulative information recorded at the baseline visit. Ethnic classification was based on the self-reported ancestry of parents and all four grandparents. Patients provided their own place of birth and that of their parents and grandparents. They were then categorised as follows: Caucasian (all ancestors of European origin); Mestizo (Latin American–born individuals with both Amerindian and European ancestry); African–Latin American (born in LATAM with at least one African ancestor, regardless of other ancestry) and Pure Amerindian (all ancestors of autochthonous origin). The following were also obtained: physician’s global assessment (PGA), disease activity assessed with the SLE Disease Activity Index 2000 (SLEDAI-2K),^23^ organ damage assessed with the SLICC/ACR Damage Index (SDI),^24^ and patient-reported outcomes including Lupus Quality of Life (LupusQoL)^25^ and Work Productivity and Activity Impairment Questionnaire.^26^ Throughout the study, a data control committee monitored the records entered, ensuring their quality.

### Patient and public involvement

Patients or the public were not involved in the design, conduct, data analysis, reporting or dissemination of this study.

### Statistical analysis

Clinical and serological markers were examined for the entire cohort and for the different patient groups. Numerical variables are reported as medians and IQRs and compared using the Kruskal-Wallis test; categorical variables are reported as frequencies (percentages) and compared using the χ^2^ or the Fisher exact tests, as appropriate. Factors associated with the development of LN and cumulative damage (SDI ≥1) were evaluated using logistic regression analysis. The results are presented as ORs and their 95% CIs. P values <0.05 were considered statistically significant. All analyses were performed with R v4.2.2.^27^

## Results

### Sociodemographic and baseline characteristics

A total of 1083 patients with SLE were enrolled into the GLADEL 2.0 cohort and included in the present analysis. These patients’ demographic characteristics are noted in table 1. Of the included patients, 89.6% were female, the median (IQR) age at diagnosis was 27 (20–35) years, and the median (IQR) disease duration at cohort entry was 66.2 (17.5–141.4) months. Most patients were of Mestizo ethnicity (65%), and 41% had a low or medium-low socioeconomic level. A total of 430 patients (39.7%) did not present with LN at enrolment (group I). Of the patients with SLE with renal involvement, 227 were from group II (prevalent inactive LN), 248 from group III (prevalent active LN) and 178 from group IV (incident active LN). Patients with LN (n=653) were significantly younger at diagnosis (median (IQR), 25 years (19.0–33.0) vs 29 years (21.0–39.0); p=0.001) and had a shorter disease duration at cohort entry (median (IQR), 58.5 months (11.7–141.2) vs 76 months (29.0–142.3); p=0.003) than patients without LN. Men had a higher LN frequency than women (72.6% vs 58.9%). A significantly higher frequency of comorbidities, such as hypertension, dyslipidaemia and secondary Cushing syndrome, was found in patients with LN (32.3%, 15.1% and 13.5%, respectively) versus patients without LN (19.4%, 7.5% and 9.1%, respectively). Disease activity, as assessed by the SLEDAI-2K, was higher among patients with LN than patients without LN (median (IQR), 8.0 (2.8–16.0) vs 2.0 (0–6.0); p*=*0.001), with a higher frequency of moderate-severe PGA in patients with LN (55.9% vs 17.4%; p=0.001). Furthermore, disease activity at cohort entry, excluding renal manifestations measured by the non-renal SLEDAI, was found to be higher in patients with LN than in patients without LN (median (IQR), 4 (2.0–7.0) vs 2 (0.0–6.0); p=0.001). However, no difference was found in cumulative damage at cohort entry between the LN and the non-LN groups (median (IQR), 0 (0–1) vs 0 (0–1); p=0.282; table 1). In online supplemental table 1, the clinical and laboratory characteristics of patients with and without LN at cohort entry are described. Within the patients with LN, proliferative LN, class III or IV, occurred in 72% of cases.

**Table 1:** Baseline characteristics of SLE patients with and without LN

Variable	Total (n=1083)	With LN (groups II–III–IV) (n=653)	Without LN (group I) (n=430)	P value*
Sex, n (%)				
Female Male	970 (89.6) 113 (10.4)	571 (58.9) 82 (72.6)	399 (41.1) 31 (27.4)	**0.004**
Age (at diagnosis), median (IQR), years	27 (20–35)	25 (19–33)	29 (21–39)	**0.001**
Age (at enrolment), median (IQR), years	35 (27–44)	33 (25–42)	38 (29–47)	**0.001**
Disease duration,† median (IQR), months	66.2 (17.6–141.4)	58.5 (11.7–141.2)	76.0 (29–142.6)	**0.003**
Education, mean (SD), years	13.3 (11-16)	13.4 (11-16)	13.1 (11-16)	0.366
Ethnic group, n (%)				0.108
African–Latin American	90 (8.3)	57 (8.8)	33 (7.7)	
Caucasian	277 (25.7)	152 (23.3)	125 (29.2)	
Mestizo	701 (65.0)	437 (67.1)	264 (61.7)	
Other	11 (1.0)	5 (0.8)	6 (1.4)	
Socioeconomic status, n (%)				0.373
Low/medium and low	438 (41.0)	272 (42.2)	166 (39.3)	
High/high, medium/high and medium	629 (59.0)	373 (57.8)	256 (60.7)	
Employment status, n (%)				**0.001**
Student	105 (10.2)	75 (12.2)	30 (7.3)	
Full-/part-time job	572 (55.6)	332 (54.0)	240 (58.1)	
Retired	45 (4.4)	17 (2.8)	28 (6.8)	
Unemployed	306 (29.8)	191 (31.1)	115 (27.8)	
Comorbidities, n (%)				
Hypertension	293 (27.2)	210 (32.3)	83 (19.4)	**0.001**
Diabetes mellitus	45 (4.2)	18 (2.8)	27 (6.3)	**0.007**
Dyslipidaemia	129 (12.1)	97 (15.1)	32 (7.5)	**0.001**
Smoking, n (%)	54 (5.1)	25 (4.0)	29 (6.8)	**0.046**
BMI, n (%)				**0.029**
Underweight	31 (3.1)	20 (3.3)	11 (2.7)	
Normal	461 (45.6)	276 (46.2)	185 (44.7)	
Overweight	333 (32.9)	209 (34.9)	124 (30)	
Obese	187 (18.5)	93 (15.6)	94 (22.7)	
Secondary Cushing’s syndrome, n (%)	127 (11.8)	88 (13.5)	39 (9.1)	**0.033**
SDI at cohort entry, median (IQR)	0 (0–1)	0 (0–1)	0 (0–1)	0.282
SDI ≥1, n (%)	405 (37.7)	253 (38.7)	152 (35.3)	0.219
SLEDAI-2K at cohort entry, median (IQR)	5 (2.0–11.0)	8 (2.8–16.0)	2 (0.0–6.0)	**0.001**
Non-renal SLEDAI at cohort entry, median (IQR)	4 (0.0–6.0)	4 (2.0–7.0)	2 (0.0–6.0)	**0.001**
PGA at cohort entry, n (%)				**0.001**
None to mild	643 (59.4)	288 (44.1)	355 (82.6)	
Moderate to severe	440 (40.6)	365 (55.9)	75 (17.4)	

* P values correspond to the Wilcoxon test, Fisher’s exact test or the χ2 test, as appropriate. Bold P values indicate statistical significance (P <0.05).

† At cohort entry.

BMI, body mass index; IQR, interquartile range; LN, lupus nephritis; PGA, physician’s global assessment; SD, standard deviation; SDI, Systemic Lupus International Collaborating Clinics/American College of Rheumatology Damage Index; SLE, systemic lupus erythematosus; SLEDAI-2K, Systemic Lupus Erythematosus Disease Activity Index.

Online supplemental figure 2 shows the distribution of cumulative damage in the different groups at cohort entry. Significant differences were found in the distribution of cumulative damage among patients in the four lupus groups. Cumulative damage (SDI ≥1) at cohort entry was found in 37.7% of all patients, 36.2% in group I patients, 41.9% in group II patients, 48.4% in group III patients and 21.3% in group IV patients (p=0.001). The highest damage values were seen in groups I and II. In online supplemental table 2a, b, the involvement of the different SDI domains in all groups is shown. Among patients with damage at cohort entry, the most frequently reported manifestations were cataracts (5.1%), cerebrovascular accidents (3.6%), proteinuria ≥3.5 g/24 hours (6.3%) and venous thrombosis (6.1%). Significant differences between groups were observed in the following domains: in group I patients (without LN), the most frequent damage domains were deforming/erosive arthritis (4%) and malignancy (1.9%). In group II patients (with prevalent inactive LN), the most frequent damage domains found were ocular (cataracts, 8.8%; retinal change or optic atrophy, 5.1%), musculoskeletal (avascular necrosis, 8.3%) and gonadal (premature gonadal failure, 6.0%). In group III patients (prevalent active LN), renal damage was the most frequent domain found, including a glomerular filtration rate <50% (9.5%), proteinuria ≥3.5 g/24 hours (19.8%) and end-stage renal disease (9.5%).

### LN characteristics

Table 2 depicts the clinical and laboratory characteristics of patients with prevalent inactive (group II), prevalent active (group III), and incident active LN (group IV). Significantly higher frequencies of proteinuria >500 mg/day (96.0%), nephrotic proteinuria (45.5%), cellular casts (37.6%) and acute renal failure (17.5%) were found in group IV patients (incident active LN). On the other hand, there was a higher frequency of chronic renal failure in groups II (11.1%) and III (12.3%). Of all patients with LN, renal biopsy was performed in 618 (94.6%). Higher frequency of diffuse proliferative LN was found in patients with prevalent active and incident active LN (123 (49.6%) and 87 (48.9%), respectively), followed by focal proliferative LN in patients with incident active LN (29.8%). In renal biopsies, the activity index had a median (IQR) of 7 (4–10) and the chronicity indexes had a median (IQR) of 2 (1–4), with no significant differences between groups. Significantly higher disease activity measured by the SLEDAI-2K was seen in patients with incident active LN (group IV; median (IQR), 16 (12–22); p*=*0.001), and patients with prevalent active LN (group III) had the highest levels of cumulative damage, with SDI ≥1 in 49.6% of patients. Table 3 in the supplemental material depicts the comparison between patients with incident active LN and patients without LN.

**Table 2:** Selected laboratory parameters in patients with LN at cohort entry

Variable	Total (n=653)	Prevalent inactive LN (group II) (n=227)	Prevalent active LN (group III) (n=248)	Incident active LN (group IV) (n=178)	P value*
Anti-dsDNA antibodies, n (%)	339 (53.8)	57 (26.0)	141 (59.2)	141 (81.5)	**<0.001**
Low C3, n (%)	532 (84.4)	168 (76.0)	208 (87.8)	156 (90.7)	**<0.001**
Low C4, n (%)	520 (82.0)	161 (73.5)	205 (86.5)	154 (90.1)	**<0.001**
Erythrocyte sedimentation rate, median (IQR)	29 (14–51)	17 (8.0–34.0)	34.5 (20.2–52.2)	45 (25.0–72.0)	**0.001**
High serum C-reactive protein, n (%)	197 (34.3)	42 (21.1)	73 (34.3)	82 (50.3)	**<0.001**
Persistent proteinuria >500 mg/day, n (%)	394 (60.8)	4 (1.8)	221 (89.1)	169 (96)	**0.001**
Nephrotic range proteinuria, n (%)	174 (27.7)	0 (0.0)	95 (39.9)	79 (45.4)	**0.001**
Cellular casts, n (%)	121 (20.5)	9 (4.4)	47 (21.4)	65 (39.4)	**<0.001**
Haematuria, n (%)	230 (37.5)	14 (6.5)	108 (47.2)	108 (63.5)	**<0.001**
Pyuria, n (%)	147 (24.3)	14 (6.6)	69 (31.1)	64 (37.9)	**<0.001**
Urinary protein/creatinine ratio, median (IQR), mg/g	216 (18.0–1360.0)	90 (9.1–245.0)	470 (19.5–1809.0)	554 (51.0–3560.0)	**0.001**
Proteinuria, median (IQR), g/24 hours	1.3 (0.5–3.4)	0.2 (0.1–0.4)	1.8 (0.8–3.8)	2.4 (1.1–4.3)	**0.001**
Serum creatinine ≥1.2 mg/dL, n (%)	136 (21.8)	23 (10.5)	67 (28.9)	46 (26.7)	**<0.001**
Acute renal failure, n (%)	58 (9)	0 (0.0)	27 (11.2)	31 (17.5)	**0.001**
Chronic renal failure, n (%)	58 (9)	25 (11.1)	30 (12.3)	3 (1.7)	**0.001**
First renal biopsy, n (%)					
Class I, minimal mesangial LN	5 (0.8)	1 (0.4)	1 (0.4)	3 (1.7)	0.323
Class II, mesangial proliferative LN	49 (7.5)	21 (9.3)	17 (6.9)	11 (6.2)	0.472
Class III, focal proliferative LN	148 (22.7)	46 (20.3)	49 (19.8)	53 (29.8)	**0.034**
Class IV, diffuse proliferative LN	293 (44.9)	83 (36.6)	123 (49.6)	87 (48.9)	**0.008**
Class V, membranous LN	121 (18.5)	34 (15.0)	44 (17.7)	43 (23.8)	0.061
Class VI, advanced sclerosing LN	2 (0.3)	0 (0.0)	2 (0.8)	0 (0.0)	0.338
Activity index, median (IQR)	7 (4-10)	8 (4-11)	7.5 (5-11)	7 (4-10)	0.665
Chronicity index, median (IQR)	2 (1-4)	2 (0–4)	2 (1-4)	2 (1-3)	0.931
Interstitial lesions, n (%)	118 (18.1)	25 (11.0)	37 (14.9)	56 (31.5)	**0.001**
Microangiopathy, n (%)	19 (2.9)	7 (3.1)	5 (27)	7 (3.9)	0.492
Antiphospholipid syndrome, n (%)	9 (1.8)	1 (0.7)	4 (2.1)	4 (2.5)	0.488
SDI ≥1, n (%)	253 (40.1)	92 (43.6)	120 (49.6)	38 (21.2)	**0.001**
SLEDAI-2K, median (IQR)	8 (2.8–16)	2 (0–4.0)	10 (6.0–16.0)	16 (12.0–22.0)	**0.001**

* P values correspond to the Kruskal-Wallis test, the Fisher’s exact test or the χ2 test, as appropriate. Bold P values indicate statistical significance (P <0.05).

C, complement component; dsDNA, double-stranded DNA; IQR, interquartile range; LN, lupus nephritis; SDI, Systemic Lupus International Collaborating Clinics/American College of Rheumatology Damage Index 2000; SLEDAI-2K, Systemic Lupus Erythematosus Disease Activity Index.

**Table 3:** Cumulative treatments in patients with prevalent and incident LN

Variable	Total (n=653)	Prevalent LN (Groups II–III) (n=475)	Incident LN (Group IV) (n=178)	P value*
Prednisone or equivalent (orally) at cohort entry, n (%)	512 (78.9)	355 (75.2)	157 (88.7)	**0.001**
Prednisone or equivalent (orally) treatment time, median (IQR), months	36 (12–96)	48 (14–100)	8 (3-24)	**0.001**
Prednisone or equivalent (orally) average dose, median (IQR), mg	33.8 (25.0–50.0)	32.5 (22.5–50.0)	40 (28.5–50.0)	**0.004**
Glucocorticoids toxicity, n (%)	139 (21.8)	113 (24.5)	26 (14.8)	**0.007**
Methylprednisolone bolus, n (%)	469 (75.9)	338 (76.3)	131 (74.9)	0.754
Antimalarials at cohort entry, n (%)	561 (86.2)	410 (86.5)	151 (85.3)	0.581
Antimalarial toxicity, n (%)	45 (7.0)	41 (8.8)	4 (2.3)	**0.003**
Azathioprine, n (%)	259 (40)	228 (48.4)	31 (17.5)	**0.001**
Cyclophosphamide IV, n (%)	360 (56.2)	296 (63.7)	64 (36.6)	**0.001**
Mycophenolate mofetil, n (%)	444 (69.1)	368 (77.8)	76 (44.7)	**0.001**
Tacrolimus, n (%)	40 (6.2)	37 (7.9)	3 (1.7)	**0.003**
Cyclosporine, n (%)	16 (2.5)	15 (3.2)	1 (0.6)	0.083
Immunoglobulin intravenous, n (%)	20 (3.1)	13 (2.8)	7 (4.0)	0.447
Belimumab, n (%)	25 (3.9)	21 (4.4)	4 (2.3)	0.255
Rituximab, n (%)	44 (7.1)	39 (8.8)	5 (2.9)	**0.009**

* P values correspond to the Wilcoxon test, the Fisher’s exact test or the χ2 test, as appropriate. Bold P values indicate statistical significance (P <0.05).

IQR, interquartile range; IV, intravenous; LN, lupus nephritis.

### Biomarkers in LN

The association between serological and urinary biomarkers within each LN group was examined. As shown in table 2, patients with incident active LN showed a higher frequency of elevated acute phase reactants (erythrocyte sedimentation rate and serum C-reactive protein), active urinary sediment (haematuria, pyuria and cylindruria) and proteinuria (urinary protein/creatinine ratio and 24-hour protein excretion). The median (IQR) urinary protein/creatinine ratio (mg/g) in group IV patients was 554 (51–3560), in group III patients was 470 (19.5–1809) and in group II patients was 90 (9.1–245; p=0.001). The median (IQR) 24-hour proteinuria was significantly higher in group IV at 2.3 g/day (1.1–4.3) when compared with groups II and III (0.2 (0.1–0.4) and 1.8 (0.8–3.8), respectively; p=0.001). Patients in group III presented a higher frequency of elevated creatinine (≥1.2 mg/dL) than patients in groups II and IV (28.9% vs 10.5% and 26.7%, respectively; p<0.001).

### Treatments in LN

Table 3 depicts the treatments used in patients with prevalent and incident LN. Prednisone or its equivalent was taken by 78.9% of the patients at cohort entry, with a median (IQR) treatment time of 36 (12–96) months and an overall glucocorticoid (GC)-associated toxicity of 21.8%, which was predominately observed in groups II and III versus group IV (24.5% vs 14.8%; p=0.007). Methylprednisolone boluses were used in 75.9% of patients, with no difference between groups. Overall, 86% of patients were using antimalarials at cohort entry, with 7% of non-users having a history of antimalarial toxicity. Cyclophosphamide was used in 56.2% of patients and mycophenolate mofetil (MMF) was used in 69.1% of patients at cohort entry. Of the 178 patients with incident active LN (group IV), 88.7% received prednisone or its oral equivalent at cohort entry, with a median (IQR) time of 8 months (3–24) and an average daily dose of 40 mg (28.5–50.0). 96% of patients with incident active LN received methylprednisolone pulses between 1 and 6 boluses, with a median (IQR) bolus dose of 500 mg (500.0–812.5). For induction, cyclophosphamide was used in 36.6% of patients, MMF in 44.7% of patients and azathioprine in 17.5% of patients. Regarding biologic therapy, belimumab was used in 2.3% and rituximab in 2.9% of patients. In terms of concomitant treatments, 78.2% of the patients received antiproteinuric/antihypertensive drugs, 19.4% of patients received anticoagulants, 16.5% of patients received statins and 32.4% of patients received vitamin D (online supplemental table 4).

### Multivariate models

After controlling for clinical and sociodemographic variables, the variables associated with the occurrence of LN were male gender (OR, 1.91; 95% CI, 1.08 to 3.38; p=0.027); Mestizo ethnicity (OR, 1.46; 95% CI, 1.02 to 2.09; p=0.040); and the presence of comorbidities (OR, 3.2; 95% CI, 2.14 to 4.79; p=0.001), thrombocytopenia (OR, 3.44; 95% CI, 2.25 to 5.26; p<0.001), anti–double-stranded DNA (anti-dsDNA) positivity (OR, 3.23; 95% CI, 1.7 to 6.13; p<0.001) and low complement levels (OR, 1.54; 95% CI, 1.05 to 2.27; p=0.028). Conversely, the factors negatively associated with LN were older age at diagnosis (OR, 0.98; 95% CI, 0.96 to 0.99; p=0.002) and discoid rash (OR, 0.53; 95% CI, 0.29 to 0.96; p=0.032). These data are depicted in table 4.

**Table 4:** Factors associated with the occurrence of LN

	Univariate model		Multivariate model	
	OR (95% CI)	P value	OR (95% CI)	P value
Age (at diagnosis), years	0.97 (0.96 to 0.98)	**<0.001**	0.98 (0.96 to 0.99)	**0.002**
Sex				
Female	Reference	–	Reference	–
Male	1.75 (1.12 to 2.73)	**0.014**	1.91 (1.08 to 3.38)	**0.027**
Ethnic group				
Caucasian	Reference	–	Reference	–
Mestizo	1.36 (1.03 to 1.80)	**0.032**	1.46 (1.02 to 2.09)	**0.040**
Socioeconomic status				
High/high medium/medium	Reference	–	Reference	–
Low/medium low	1.13 (0.87 to 1.47)	0.360	1.29 (0.91 to 1.82)	0.147
Comorbidities	2.18 (1.62 to 2.92)	**<0.001**	3.2 (2.14 to 4.79)	**<0.001**
Fever	1.54 (1.18 to 2.00)	**0.002**	1.17 (0.82 to 1.68)	0.382
Malar rash	1.16 (0.90 to 1.51)	0.253	1.28 (0.90 to 1.84)	0.173
Discoid rash	0.51 (0.31 to 0.82)	**0.005**	0.53 (0.29 to 0.96)	**0.037**
Oral ulcers	0.91 (0.71 to 1.18)	0.492	0.73 (0.51 to 1.03)	0.073
Arthritis	0.77 (0.55 to 1.07)	0.123	0.67 (0.43 to 1.05)	0.080
Pleuritis/pericarditis	0.82 (0.64 to 1.07)	0.142	0.77 (0.52 to 1.13)	0.178
Leucopenia	1.09 (0.84 to 1.41)	0.513	0.95 (0.64 to 1.39)	0.784
Lymphopenia	0.76 (0.56 to 1.03)	0.072	0.73 (0.49 to 1.07)	0.107
Thrombocytopenia	3.63 (2.64 to 5.00)	**<0.001**	3.44 (2.25 to 5.26)	**<0.001**
Anti-dsDNA positivity	3.06 (1.82 to 5.14)	**<0.001**	3.23 (1.7 to 6.13)	**<0.001**
Low complement	1.86 (1.36 to 2.54)	**<0.001**	1.54 (1.05 to 2.27)	**0.028**

Bold P values indicate statistical significance (P <0.05).

CI, confidence interval; dsDNA, double-stranded DNA; LN, lupus nephritis; OR, odds ratio.

After adjustment for renal involvement, sociodemographic characteristics, disease activity and treatments (table 5), the variables associated with cumulative damage (SDI ≥1) were older age at diagnosis (OR, 1.02; 95% CI, 1.01 to 1.03; p=0.014), longer disease duration (OR, 1.01; 95% CI, 1.01 to 1.02; p<0.001), cumulative prednisone dose or equivalent (OR, 1.01; 95% CI, 1.01 to 1.02; p=0.005) and cyclophosphamide use (OR, 2.16; 95% CI, 1.48 to 3.16; p<0.001). Prevalent active LN (group III) was significantly associated with cumulative damage in the univariate analysis (OR, 1.77; 95% CI, 0.97 to 1.86; p<0.001), but this significance was no longer present in the multivariate analysis (OR, 1.29; 95% CI, 0.76 to 2.19; p=0.344).

**Table 5:** Factors associated with damage accrual at cohort entry

	Univariate model		Multivariate model	
	OR (95% CI)	P value	OR (95% CI)	P value
Group I (without LN)	Reference	–	Reference	–
Group II (prevalent inactive LN)	1.35 (0.96 to 1.88)	0.085	0.71 (0.45 to 1.11)	0.137
Group III (prevalent active LN)	1.77 (0.97 to 1.86)	<0.001	1.29 (0.76 to 2.19)	0.344
Age (at diagnosis), years	1.00 (0.99 to 1.01)	0.600	1.02 (1.01 to 1.03)	**0.014**
Disease duration, months	1.01 (1.00 to 1.01)	<0.001	1.01 (1.01 to 1.02)	**<0.001**
Sex				
Female	Reference	–	Reference	–
Male	1.09 (0.72 to 1.63)	0.691	1.26 (0.73 to 2.18)	0.396
Education, years	0.96 (0.93 to 0.99)	0.011	0.96 (0.91 to 1.00)	0.052
Ethnic group				
Caucasian	Reference	–	Reference	–
African–Latin American	1.22 (0.75 to 1.98)	0.431	1.65 (0.91 to 3.01)	0.10
Mestizo	0.97 (0.91 to 1.62)	0.844	1.11 (0.78 to 1.57)	0.572
Socioeconomic status				
High/high medium/medium	Reference	–	Reference	–
Low/medium low	1.13 (0.88 to 1.46)	0.342	0.99 (0.7 to 1.4)	0.937
Comorbidities	2.41 (1.85 to 3.15)	<0.001	1.69 (1.20 to 2.39)	**0.003**
SLEDAI-2K at cohort entry ≥6	0.87 (0.68 to 1.12)	0.274	1.03 (0.70 to 1.53)	0.871
Prednisone or equivalent accumulated dose, g	1.01 (1.01 to 1.01)	<0.001	1.01 (1.01 to 1.02)	**0.005**
Antimalarials	1.26 (0.62 to 2.55)	0.518	1.46 (0.41 to 5.18)	0.556
Cyclophosphamide intravenous	2.44 (1.87 to 3.17)	<0.001	2.16 (1.48 to 3.16)	**<0.001**
Mycophenolate mofetil	1.36 (1.06 to 1.75)	0.017	1.01 (0.64 to 1.35)	0.707

Bold P values indicate statistical significance (P <0.05).

CI, confidence interval; LN, lupus nephritis; OR, odds ratio; SLEDAI-2K, Systemic Lupus Erythematosus Disease Activity Index.

## Discussion

This is the first comprehensive report of the baseline characteristics of the large, multicentre, multiethnic LATAM cohort, GLADEL 2.0. This cohort includes 1083 patients from 10 countries to ensure that these data are broadly representative of the region’s diverse patient population. We found a clear female predominance with a mean age of 35 years at cohort entry. The education level and socioeconomic status of patients with or without LN were comparable, and that was also the case for ethnicity; however, we found that more than half of the patients in the entire cohort were Mestizo. On the other hand, patients with LN had a shorter disease duration at cohort entry than those without it. Similar data were found in the original GLADEL cohort; of the 1214 patients with lupus who were included, 90% were women and 44% of the cohort were Mestizos, and renal disease was found to be significantly more prevalent in Mestizo patients and African–Latin American patients than in White patients.^1^ Furthermore, in the US PROFILE cohort, Hispanic patients (individuals tracing their origin to a Spanish-speaking country, many of them with Native American and White ancestry, like the Mestizos in LATAM) were more likely than patients of other ethnic groups to meet ACR renal criteria.^28^ In a similar sense, the RELESSER cohort showed that Latin American Hispanic patients experienced their first SLE symptom 4 years earlier, were diagnosed at a younger age, more frequently suffered from nephritis and had higher severity indices than European Caucasian patients.^29^ These findings reinforce the imperative to accurately characterise the clinical expression of lupus in Latin American populations. Table 5 in the supplemental material presents the comparative clinical characteristics across different cohorts of patients with SLE.

Significant differences were found in the specific SDI domains between the groups. Two-thirds of the patients in the current GLADEL 2.0 cohort had no cumulative damage at cohort entry, with a higher frequency of patients without cumulative damage in the incident LN group (group IV). Patients with higher SDI values were seen in group I (without LN) and group II (prevalent inactive LN). This may be related to the treatments patients had received, especially with glucocorticoids (GCs). Previously, Apostolopoulos *et al* have demonstrated that GC use in lupus is associated with the accrual of irreversible organ damage, independent of disease activity,^30^ both in domains traditionally associated with GC-induced damage such as cataracts, osteoporotic fracture, avascular necrosis and diabetes mellitus, but also in domains not previously associated with GC-induced damage such as renal, pulmonary, gastrointestinal, skin, premature gonadal failure and malignancy.^30^,^33^

Patients with incident LN (group IV) were found to be younger at diagnosis and more frequently male. While these patients had higher disease activity and a higher frequency of comorbidities at cohort entry, no greater cumulative damage was found; this probably reflects the fact that this group of patients was younger and had a shorter disease duration than the patients without LN. These findings are similar to those described by Hanly *et al*, who found that of 1827 patients, 38.3% had LN and they were younger; more frequently male; and more frequently of African, Asian or Hispanic race/ethnicity.^34^ Similar findings were described in the SLICC Inception cohort, where they found that age significantly influenced the likelihood of damage accrual.^35^ Likewise, in the Hopkins lupus cohort, damage accrual was higher in patients who were older, male, African-American, had low income, had low education, were hypertensive, lupus anticoagulant positive and/or had elevated proteinuria.^36^ Patients with incident LN showed higher acute-phase reactant levels, more frequent anti-dsDNA positivity and lower complement levels at cohort entry. They also exhibited high rates of persistent proteinuria >500 mg/day, nephrotic-range proteinuria, active urinary sediment, acute renal failure and a predominance of class III focal proliferative LN on renal biopsy. These groups of patients have less accrual damage (SDI ≥1) and more active disease as measured by SLEDAI-2K.

The use of biomarkers to identify patient subsets allows monitoring of treatment response and disease activity detection. Proteinuria, urine protein/creatinine ratio, creatinine clearance, anti-dsDNA and complement levels are currently used LN laboratory markers, but they lack specificity and sensitivity for identifying renal activity and damage.^37^ Among them, in the GLADEL 2.0 cohort, proteinuria >500 mg/day was the most relevant biomarker associated with LN activity. In addition, chronic renal failure was present in 10% of patients with prevalent active and inactive LN at cohort entry. In patients with LN who underwent renal biopsy, the most frequent presentation found was diffuse proliferative LN in almost half of the patients, followed by focal proliferative LN. Less damage accrual and higher disease activity were found in patients with incident LN compared with patients with prevalent LN (active and inactive). These findings are consistent with the data presented by Malvar *et al* in a cohort of Argentinean LN patients.^38^

The treatment of SLE and of LN remains a challenge. Patients can progress to end-stage renal disease without effective control of LN activity and experience a deterioration of their quality of life and increased mortality. This is evident in LATAM, where accessibility to the health system and effective treatments, especially biologic therapies, are impacted by socioeconomic determinants of health and the diverse health systems; this is particularly the case for indigenous populations.^39^ The different therapeutic strategies for prevalent and incident patients with LN were compared. Antimalarials were used in almost all patients, with a low incidence of toxicity. GCs remain a cornerstone in the management of these patients, mostly in underdeveloped countries. Oral prednisone was used in almost all patients, with a higher frequency and higher average dose in incident LN. We also observed that methylprednisolone pulses were indicated in more than 70% of patients in both groups; however, they did not preclude the development of associated toxicity, which occurred in 20% of the patients. Immunosuppressants are the mainstay in the management of these patients. We observed a decrease over time in the use of cyclophosphamide, while in parallel, there was an increase in the use of MMF. In contrast with the original GLADEL cohort, less oral GCs (79% vs 92%) and more antimalarials (86% vs 75%) were used. In the original GLADEL cohort, immunosuppressants were used in 47% of patients, with intravenous cyclophosphamide predominating, followed by azathioprine; in the current cohort, MMF was the most indicated immunosuppressant.^1^ The use of biologics in the present cohort remains a challenge, although they were used in some patients with LN who did not respond or who relapsed; this represented slightly more than 10% of the patients.

In the evaluation of factors associated with the presence of LN at cohort entry, a significant association was observed with younger age at diagnosis, male sex, Mestizo ethnicity and the presence of comorbidities. Thrombocytopenia, positive anti-dsDNA and low complement at cohort entry were also associated with the presence of LN. These data are consistent with those observed in the original GLADEL cohort.^11^ In addition, we observed that discoid rash was negatively associated with renal involvement in our patients, which is consistent with data from the original GLADEL cohort.^40^

In the GLADEL 2.0 cohort, cumulative damage at cohort entry was observed in more than one-third of the patients, and it was unrelated to the presence of LN. Adjusted for confounders, damage was found to be associated with older age at diagnosis, longer disease duration, presence of comorbidities and a history of higher cumulative GC dose and cyclophosphamide use. In this sense, in the Asia Pacific Lupus Collaboration, 48% of patients had organ damage at enrolment and, after a median follow-up period of 795 days, 14% had accumulated further irreversible organ damage.^41^ In addition, they found that the history of active LN, age and cumulative prednisone dose was associated with an increased risk of irreversible damage. Assessing damage at the beginning of follow-up is important because of its impact on the course of the disease. Thus, Bruce *et al* studied the impact of damage accrual in the SLICC Inception cohort; in this cohort of 1722 patients, having damage at cohort entry was associated with a 46% increase in mortality (hazard ratio, 1.46; 95% CI 1.18 to 1.81; per SDI point).^35^

Our current study has several limitations. First, GLADEL 2.0 is an observational cohort with well-defined inclusion and exclusion criteria but does not have local monitors to evaluate the information provided; therefore, a centralised data control committee has been established to ensure data quality and avoid errors. Second, haematological, chemical and immunological laboratory determinations are being performed locally, thus there may be technical differences between the different LATAM centres. Third, a self-reported ethnicity definition (of both parents and all four grandparents) was used, which may have led to some misclassification errors. Fourth, regarding treatments, a small number of patients with SLE received biological therapy in all cohorts. In addition, due to the retrospective nature of the data, detailed information on treatment access, adherence, delays in treatment initiation and treatment gaps prior to cohort entry was not available. Finally, an exact temporality between determinants and outcomes is not available when examining cumulative baseline data.

The greatest strength of this study is that it represents the variability of lupus in LATAM, given that centres from 10 countries were included. This large, heterogeneous sample enhances the generalisability of our findings, offering insights that are applicable across different ethnic groups and socioeconomic backgrounds within LATAM. It fills an important knowledge gap by providing detailed baseline data that reflect the unique epidemiological and socioeconomic characteristics of lupus in LATAM. The study provides an in-depth analysis of patients with SLE with and without LN, revealing important associations between age at diagnosis, male sex and Mestizo ethnicity and the development of LN. These findings are critical since, as is well known, LN is a major determinant of morbidity and mortality in SLE and understanding its baseline characteristics can directly influence treatment decisions and prognosis. Another major strength of this cohort is the possibility of future biomarker research through the centralised analysis of urinary and serological samples, which can be analysed in connection with the results of the renal biopsy since these are available in more than 90% of patients with LN. This resource may prove invaluable for the validation of new biomarkers that have the potential to improve diagnosis, follow-up and risk stratification for patients with LN. This communication lays the ground for further studies that will integrate these biomarkers into clinical practice, potentially transforming the way LN is treated in LATAM.

In summary, the results of this study provide a detailed analysis of the baseline characteristics of patients with and without LN from a large multicentre LATAM cohort, GLADEL 2.0. In this cohort, Mestizo ethnicity continues to have a significant association with renal involvement. In addition, renal involvement was associated with younger age at diagnosis, male sex, presence of comorbidities, thrombocytopenia, anti-dsDNA antibodies and low complement. Cumulative damage was associated with older age at diagnosis, longer disease duration, cyclophosphamide use and higher cumulative corticosteroid dose. Furthermore, we will be able to assess the diagnostic and prognostic performance of new biomarkers in patients with LN. Efforts are now underway to examine the centralised urinary and serological databank; such data may improve our ability to diagnose and treat LN earlier than at the present time, potentially improving patient outcomes.

## Supplementary material

10.1136/lupus-2025-001878 online supplemental file 1

## Data Availability

The datasets generated and/or analysed during the current study are available from the corresponding author on reasonable request.
