# Supplementary material for: Baseline characteristics of patients with SLE with and without lupus nephritis from the Latin American multiethnic GLADEL 2.0 cohort
Source: Lupus Sci Med. 2026 Apr 20;13(1):e001878. doi: 10.1136/lupus-2025-001878 (PMC13110673; doi:10.1136/lupus-2025-001878)
Supplement: online supplemental file 1 [file lupus-13-1-s001.docx]

**Supplementary Material**

**Baseline Characteristics of Patients With Systemic Lupus Erythematosus With and Without Lupus Nephritis From the Latin American Multi-Ethnic GLADEL 2.0 Cohort**

**Supplemental Table 1: Clinical and Laboratory Characteristics of Patients With and Without Lupus Nephritis (LN)**

| **Variable** | **Total**  **(N=1083)** | **Without LN**  **(Group I)**  **(N=430)** | **With LN**  **(Groups II, III, and IV)**  **(N=653)** | ***p-*value^a^** |
| --- | --- | --- | --- | --- |
| **Fever, n (%)** | 446 (41.3) | 153 (35.7) | 293 (44.9) | **0.002** |
| **Malar rash, n (%)** | 657 (60.7) | 254 (59.1) | 403 (61.8) | 0.374 |
| **Discoid rash, n (%)** | 88 (8.1) | 49 (11.4) | 39 (6.0) | **0.002** |
| **Photosensitivity, n (%)** | 666 (61.8) | 274 (63.7) | 392 (60.6) | 0.306 |
| **Bullous lupus, n (%)** | 12 (1.1) | 1 (0.2) | 11 (1.7) | **0.034** |
| **Hypertrophic lupus, n (%)** | 5 (0.5) | 1 (0.2) | 4 (0.6) | 0.654 |
| **Lupus panniculitis, n (%)** | 14 (1.3) | 8 (1.9) | 6 (0.9) | 0.271 |
| **Lupus tumidus, n (%)** | 1 (0.1) | 1 (0.2) | 0 (0.0) | 0.398 |
| **Lupus pernio, n (%)** | 14 (1.3) | 8 (1.9) | 6 (0.9) | 0.271 |
| **Toxic epidermal necrolysis, n (%)** | 3 (0.3) | 1 (0.2) | 2 (0.3) | 1.000 |
| **Acute cutaneous lupus, n (%)** | 542 (50.2) | 215 (50.0) | 327 (50.3) | 0.950 |
| **Oral/nasopharyngeal ulcers, n (%)** | 474 (44.1) | 193 (44.9) | 281 (43.6) | 0.707 |
| **Alopecia, n (%)** | 705 (65.3) | 274 (63.7) | 431 (66.3) | 0.396 |
| **Arthritis, n (%)** | 874 (80.8) | 357 (83) | 517 (79.3) | 0.135 |
| **Pleuritis, n (%)** | 278 (25.8) | 84 (19.6) | 194 (29.8) | **0.001** |
| **Pericarditis, n (%)** | 193 (17.9) | 51 (11.9) | 142 (21.9) | **0.001** |
| **Persistent proteinuria, n (%)** | 609 (56.4) | 0 (0.0) | 609 (93.7) | **0.001** |
| **Cellular casts, n (%)** | 273 (26.6) | 0 (0.0) | 273 (45.7) | **0.001** |
| **Class II or V renal biopsy, n (%)** | 177 (16.7) | 0 (0.0) | 177 (27.9) | **0.001** |
| **Class III or IV renal biopsy, n (%)** | 460 (43.4) | 0 (0.0) | 460 (72.3) | **0.001** |
| **Delirium, n (%)** | 17 (1.6) | 6 (1.4) | 11 (1.7) | 0.806 |
| **Psychosis, n (%)** | 37 (3.4) | 15 (3.5) | 22 (3.4) | 1.000 |
| **Seizures, n (%)** | 55 (5.1) | 19 (4.4) | 36 (5.5) | 0.480 |
| **Mononeuritis multiplex, n (%)** | 11 (27) | 8 (1.9) | 3 (0.5) | **0.031** |
| **Myelitis, n (%)** | 7 (0.6) | 4 (0.9) | 3 (0.5) | 0.446 |
| **Peripheral or cranial neuropathy, n (%)** | 40 (3.7) | 23 (5.4) | 17 (2.6) | **0.022** |
| **Acute confusional state, n (%)** | 19 (1.8) | 8 (1.9) | 11 (1.7) | 0.818 |
| **Hemolytic anemia, n (%)** | 129 (12) | 55 (12.9) | 74 (11.5) | 0.503 |
| **Leukopenia, n (%)** | 494 (46.4) | 205 (48.5) | 289 (45.0) | 0.286 |
| **Lymphopenia, n (%)** | 580 (54.5) | 223 (52.7) | 357 (55.7) | 0.346 |
| **Thrombocytopenia, n (%)** | 246 (23.1) | 112 (26.4) | 134 (20.9) | **0.045** |
| **ANA positivity, n (%)** | 1061 (99.2) | 425 (98.8) | 636 (99.4) | 0.497 |
| **Anti-dsDNA positivity, n (%)** | 818 (78.0) | 268 (64.6) | 550 (86.8) | **0.001** |
| **Anti-Sm positivity, n (%)** | 312 (34.5) | 107 (28.2) | 205 (39.0) | **0.001** |
| **Lupus anticoagulant positivity, n (%)** | 142 (16.5) | 67 (18.9) | 75 (14.9) | 0.136 |
| **Anticardiolipin antibodies positivity, n (%)** | 178 (19.7) | 85 (22.9) | 93 (17.5) | 0.050 |
| **Anti-B2GPI antibodies positivity, n (%)** | 89 (12.2) | 49 (16.4) | 40 (9.2) | **0.004** |
| **VDRL false positivity, n (%)** | 35 (4.5) | 15 (4.7) | 20 (4.3) | 0.861 |
| **Low C3, n (%)** | 810 (76.6) | 266 (63.3) | 544 (85.4) | **0.001** |
| **Low C4, n (%)** | 817 (77.6) | 281 (66.9) | 536 (84.7) | **0.001** |
| **Low CH50, n (%)** | 80 (26.3) | 26 (19.4) | 54 (31.8) | **0.018** |
| **Coombs positivity, n (%)** | 183 (24.5) | 64 (21.4) | 119 (26.6) | 0.118 |

ANA, antinuclear antibodies; C, complement component; CH50, total complement; VDRL, Venereal Disease Research Laboratory.

^a^*p*-values correspond to the Fisher's exact test.

**Supplemental Table 2a: Damage in SLE Groups at Cohort Entry**

| **SDI total, n (%)** | **Total (N=1073)** | **Without LN**  **(Group I)**  **(N=420)** | **Prevalent inactive LN**  **(Group II)**  **(N=227)** | **Prevalent active LN**  **(Group III)**  **(N=242)** | **Incident active LN**  **(Group IV)**  **(N=178)** | ***p-*value^a^** |
| --- | --- | --- | --- | --- | --- | --- |
| 0 | 668 (62.3) | 268 (63.8) | 132 (58.1) | 128 (51.6) | 140 (78.7) | **0.001** |
| 1 | 222 (20.7) | 82 (19.5) | 44 (20.2) | 75 (31) | 21 (12.3) |  |
| 2 | 96 (8.9) | 32 (7.6) | 25 (11.5) | 28 (11.6) | 11 (6.4) |  |
| 3 | 49 (4.6) | 21 (5.0) | 15 (6.9) | 9 (3.7) | 4 (2.3) |  |
| ≥4 | 38 (3.5) | 17 (4.0) | 11 (5.1) | 8 (3.3) | 2 (1.2) |  |

LN, lupus nephritis; SDI, Systemic Lupus International Collaborating Clinics/American College of Rheumatology Damage Index; SLE, systemic lupus erythematosus.

^a^*p-*values correspond to the Chi-Square Test.

**Supplemental Table 2b: SDI Items in All Groups at Cohort Entry**

| **Variable, n (%)** | **Total (N=1051)** | **Without LN**  **(Group I)**  **(N=420)** | **Prevalent inactive LN**  **(Group II)**  **(N=219)** | **Prevalent active LN**  **(Group III)**  **(N=238)** | **Incident active LN**  **(Group IV)**  **(N=174)** | ***p-*value^a^** |
| --- | --- | --- | --- | --- | --- | --- |
| **Cataract** | 54 (5.1) | 21 (5.0) | 19 (8.8) | 13 (5.4) | 1 (0.6) | **0.002** |
| **Retinal change or optic atrophy** | 23 (2.2) | 6 (1.4) | 11 (5.1) | 5 (2.1) | 1 (0.6) | **0.017** |
| **Cognitive impairment** | 22 (2.1) | 11 (2.6) | 5 (2.3) | 5 (2.1) | 1 (0.6) | 0.468 |
| **Seizures requiring therapy for 6 months** | 19 (1.8) | 10 (2.4) | 6 (2.8) | 3 (1.2) | 0 (0.0) | 0.105 |
| **Cerebrovascular accident** | 38 (3.6) | 16 (3.8) | 9 (4.1) | 8 (3.3) | 5 (2.9) | 0.926 |
| **Cranial or peripheral neuropathy (excluding optic)** | 23 (2.2) | 12 (2.9) | 5 (2.3) | 4 (1.7) | 2 (1.2) | 0.631 |
| **Transverse myelitis** | 5 (0.5) | 3 (0.7) | 0 (0.0) | 2 (0.8) | 0 (0.0) | 0.549 |
| **Estimated or measured glomerular filtration rate <50%** | 42 (4.0) | 0 (0.0) | 14 (6.4) | 23 (9.5) | 5 (2.9) | **0.001** |
| **Proteinuria ≥ 3.5 g/24h** | 66 (6.3) | 0 (0.0) | 1 (0.5) | 48 (19.8) | 17 (9.9) | **0.001** |
| **End-stage renal disease** | 11 (1.0) | 0 (0.0) | 4 (1.8) | 6 (2.5) | 1 (0.6) | **0.003** |
| **Pulmonary hypertension** | 16 (1.5) | 6 (1.4) | 6 (2.8) | 2 (0.8) | 2 (1.2) | 0.421 |
| **Pulmonary fibrosis** | 19 (1.8) | 13 (3.1) | 4 (1.8) | 1 (0.4) | 1 (0.6) | 0.050 |
| **Shrinking lung** | 16 (1.5) | 7 (1.7) | 4 (1.8) | 5 (2.1) | 0 (0.0) | 0.286 |
| **Pleural fibrosis** | 2 (0.2) | 0 (0.0) | 0 (0.0) | 1 (0.4) | 1 (0.6) | 0.265 |
| **Pulmonary infarction** | 9 (0.9) | 4 [27] | 1 (0.5) | 4 (1.7) | 0 (0.0) | 0.346 |
| **Angina or coronary artery bypass** | 4 (0.4) | 3 (0.7) | 0 (0.0) | 1 (0.4) | 0 (0.0) | 0.696 |
| **Myocardial infarction** | 6 (0.6) | 4 (1.0) | 0 (0.0) | 1 (0.4) | 1 (0.6) | 0.622 |
| **Cardiomyopathy (ventricular dysfunction)** | 6 (0.6) | 3 (0.7) | 2 (0.9) | 1 (0.4) | 0 (0.0) | 0.799 |
| **Valvular disease** | 10 (0.9) | 2 (0.5) | 6 (2.8) | 1 (0.4) | 1 (0.6) | **0.039** |
| **Pericarditis** | 16 (1.5) | 5 (1.2) | 4 (1.8) | 3 (1.2) | 4 (2.3) | 0.658 |
| **Claudication for 6 months** | 2 (0.2) | 1 (0.2) | 1 (0.5) | 0 (0.0) | 0 (0.0) | 0.815 |
| **Minor tissue loss** | 6 (0.6) | 3 (0.7) | 1 (0.5) | 2 (0.8) | 0 (0.0) | 0.842 |
| **Significant tissue loss ever (eg, loss of digit or limb)** | 3 (0.3) | 1 (0.2) | 0 (0.0) | 2 (0.8) | 0 (0.0) | 0.363 |
| **Venous thrombosis with swelling, ulceration, or venous stasis** | 64 (6.1) | 23 (5.5) | 18 (8.3) | 15 (6.2) | 8 (4.7) | 0.465 |
| **Infarction or resection of bowel below duodenum, spleen, liver, or gallbladder ever, for any cause** | 12 (1.1) | 5 (1.2) | 4 (1.8) | 2 (0.8) | 1 (0.6) | 0.716 |
| **Mesenteric insufficiency** | 1 (0.1) | 1 (0.2) | 0 (0.0) | 0 (0.0) | 0 (0.0) | 1.000 |
| **Chronic peritonitis** | 1 (0.1) | 0 (0.0) | 0 (0.0) | 0 (0.0) | 1 (0.6) | 0.163 |
| **Stricture or upper gastrointestinal tract surgery ever** | 2 (0.2) | 1 (0.2) | 0 (0.0) | 1 (0.4) | 0 (0.0) | 1.000 |
| **Muscle atrophy or weakness** | 17 (1.6) | 10 (2.4) | 4 (1.8) | 2 (0.8) | 1 (0.6) | 0.360 |
| **Deforming or erosive arthritis** | 30 (2.9) | 17 (4.0) | 8 (3.7) | 5 (2.1) | 0 (0.0) | **0.017** |
| **Osteoporosis with fracture or vertebral collapse** | 14 (1.3) | 9 (2.1) | 4 (1.8) | 1 (0.4) | 0 (0.0) | 0.091 |
| **Avascular necrosis** | 41 (3.9) | 16 (3.8) | 18 (8.3) | 5 (2.1) | 2 (1.2) | **0.002** |
| **Osteomyelitis** | 5 (0.5) | 3 (0.7) | 2 (0.9) | 0 (0.0) | 0 (0.0) | 0.393 |
| **Scarring chronic alopecia** | 19 (1.8) | 11 (2.6) | 4 (1.8) | 4 (1.7) | 0 (0.0) | 0.153 |
| **Extensive scarring or panniculus other than scalp and pulp space** | 11 (1.0) | 7 (1.7) | 3 (1.4) | 0 (0.0) | 1 (0.6) | 0.154 |
| **Skin ulceration (excluding thrombosis) for >6 months** | 4 (0.4) | 3 (0.7) | 0 (0.0) | 0 (0.0) | 1 (0.6) | 0.434 |
| **Premature gonadal failure** | 28 (2.7) | 9 (2.1) | 13 (6.0) | 5 (2.1) | 1 (0.6) | **0.010** |
| **Diabetes** | 33 (3.1) | 21 (5) | 4 (1.9) | 4 (1.7) | 4 (2.3) | 0.062 |
| **Malignancy** | 10 (1.0) | 8 (1.9) | 2 (0.9) | 0 (0.0) | 0 (0.0) | **0.041** |

LN, lupus nephritis; SDI, Systemic Lupus International Collaborating Clinics/American College of Rheumatology Damage Index.

^a^ *p*-values correspond to the Wilcoxon Test, the Fisher's Exact Test, or the Chi-Square Test, as appropriate.

**Supplemental Table 3: Comparison of patients with active incident lupus nephritis (LN) vs. without it**

| **Variable** | **Total (n=608)** | **Without LN**  **(Group I) (n=430)** | **Incident LN**  **(Group IV) (n=178)** | ***p*^a^ value** |
| --- | --- | --- | --- | --- |
| **Age (at diagnosis) years, median (Q1-Q3)** | 29 (21-38) | 29 (21-39) | 27.5 (22-36) | 0.346 |
| **Age (at enrollment) years, median (Q1-Q3)** | 36 (28-44) | 38 (29-47) | 30.5 (23.2-39) | **0.001** |
| **Disease duration (in months), median (Q1-Q3)** | 48.5 (8.6-125.2) | 76.0 (29-141.3) | 3.2 (0.7-28.1) | **0.001** |
| **Sex, n(%)** |  |  |  | **0.001** |
| Female | 544 (89.5) | 399 (92.8) | 145 (81.5) |  |
| Male | 64 (10.5) | 31 (7.2) | 33 (18.5) |  |
| **Education (in years), median (Q1-Q3)** | 13 (11-16) | 13 (11-16) | 12 (11-15) | 0.330 |
| **Ethnic group, n(%)** |  |  |  | 0.122 |
| Afro-Latin American | 50 (8.3) | 33 (7.7) | 17 (9.6) |  |
| Caucasian | 161 (26.6) | 125 (29.2) | 36 (20.2) |  |
| Mestizo | 387 (63.9) | 264 (61.7) | 123 (69.1) |  |
| Other | 8 (1.3) | 6 (1.4) | 2 (1.1) |  |
| **Socioeconomic status, n(%)** |  |  |  | **0.024** |
| Low/medium and low | 253 (42.3) | 166 (39.3) | 87 (49.4) |  |
| High/high, medium/high, and medium | 345 (57.7) | 256 (60.7) | 89 (50.6) |  |
| **Employment status, n(%)** |  |  |  | **0.001** |
| Student | 56 (9.6) | 30 (7.3) | 26 (15.3) |  |
| Full/part time job | 334 (57.3) | 240 (58.1) | 94 (55.3) |  |
| Retired | 29 (5.0) | 28 (6.8) | 1 (0.6) |  |
| Unemployed | 164 (28.1) | 115 (27.8) | 49 (28.8) |  |
| **Comorbidities, n(%)** |  |  |  |  |
| Hypertension | 132 (21.8) | 83 (19.4) | 49 (27.5) | **0.031** |
| Diabetes mellitus | 33 (5.4) | 27 (6.3) | 6 (3.4) | 0.172 |
| Dyslipidemia | 58 (9.7) | 32 (7.5) | 26 (14.9) | **0.009** |
| **Smoking, n%** | 39 (6.5) | 29 (6.8) | 10 (5.8) | 0.855 |
| **BMI, n(%)** |  |  |  | **0.038** |
| Underweight | 17 (3.0) | 11 (2.7) | 6 (3.9) |  |
| Normal | 248 (43.6) | 185 (44.7) | 63 (40.6) |  |
| Overweight | 187 (32.9) | 124 (30.0) | 63 (40.6) |  |
| Obese | 117 (20.6) | 94 (22.7%) | 23 (14.8) |  |
| **Secondary Cushing’s syndrome, n(%)** | 56 (9.2) | 39 (9.1) | 17 (9.6) | 0.878 |
| **SDI** >=1**, n(%)** | 190 (32.1) | 152 (36.2) | 38 (22.2) | **0.001** |
| **SLEDAI-2K, median (Q1-Q3)** | 4 (1-11) | 2 (0-6) | 16 (12-22) | **0.001** |
| **SLEDAI-2K renal, median (Q1-Q3)** | 4 (0-8) | 2 (0-6) | 8 (4-11) | **0.001** |
| **PGA, n(%)** |  |  |  | **0.001** |
| None to mild | 377 (62.0) | 355 (82.6) | 22 (12.4) |  |
| Moderate to severe | 231 (38.0) | 75 (17.4) | 156 (87.6) |  |

^a^*p-*value corresponding to the Wilcoxon test or Fisher's exact test or Chi-square test, as appropriate.

**Supplemental Table 4: Therapeutic Strategies Used in Patients With Incident Lupus Nephritis (LN)**

| **Variable** | **Incident active LN**  **(Group IV)**  **(N=178)** |
| --- | --- |
| **Prednisone or equivalent (orally) at cohort entry, n (%)** | 157 (88.7) |
| **Prednisone or equivalent treatment time, median (IQR), months** | 8 (3-24) |
| **Prednisone or equivalent (orally) - average dose, median (IQR), grams** | 40 (28.5-50) |
| **Glucocorticoids toxicity, n (%)** | 26 (14.8) |
| **Methylprednisolone bolus, n (%)** | 131 (74.9) |
| **Total number of boluses, mean (SD)** | 3.1 (0.9) |
| **Bolus dose, mean (SD), mg/bolus** | 616.4 (231.8) |
| **Antimalarials at cohort entry, n (%)** | 151 (85.3) |
| **Antimalarials - toxicity, n (%)** | 4 (2.3) |
| **Azathioprine, n (%)** | 31 (17.5) |
| **IV cyclophosphamide, n (%)** | 64 (36.6) |
| **Mycophenolate mofetil, n (%)** | 76 (44.7) |
| **Tacrolimus, n (%)** | 3 (1.7) |
| **Cyclosporine, n (%)** | 1 (0.6) |
| **Immunoglobulin IV, n (%)** | 7 (4.0) |
| **Belimumab, n (%)** | 4 (2.3) |
| **Rituximab, n (%)** | 5 (2.9) |
| **Anticoagulants, n (%)** | 34 (19.4) |
| **Antihypertensive agents, n (%)** | 52 (29.9) |
| **Antiproteinuric agents, n (%)** | 85 (48.3) |
| **Vitamin D, n (%)** | 57 (32.4) |
| **Statins, n (%)** | 29 (16.5) |

IQR, interquartile range; IV, intravenous; SD, standard deviation.

**Supplemental Table 5: Patient Characteristics Across Studies**

|  | **GLADEL 2.0**  **(N=1083)** | **GLADEL 1.0 (N=1214) [1]** | **RELESSER  (N=3490) [30]** | **Asia Pacific Lupus Collaboration (N=1735) [42]** | **SLICC Inception Cohort (N=1722) [36]** | **PROFILE (N=555) [29]** | **Hopkins lupus cohort**  **(N=2054) [37]** | **Apostolopoulos (N=1707) [31]** | **Malvar (N=110) [39]** |
| --- | --- | --- | --- | --- | --- | --- | --- | --- | --- |
| **Sex, n (%)** |  |  |  |  |  |  |  |  |  |
| Female | 970 (89.6) | 1091 (89.9) | 3157 (90) | (93) | 1536 (89.2) | (86.2-96.2)^a^ | 1899 (92) | 1591 (93.2) | 89 (81) |
| Male | 113 (10.4) | 123 (10.1) | 333 (10) | (7) | 186 (10.8) | (3.8-13.8)^a^ | 155 (8) | 116 (6.8) | 21 (19) |
| **Race/Ethnicity, n (%)** |  |  |  |  |  |  |  |  |  |
| African origin | 90 (8.3) | 152 (12.5) | – | – | 280 (16.3) | 217 (39.1) | 761 (37) | – | – |
| Caucasian | 277 (25.7) | 507 (41.8) | 3305 (95) | (~10) | 830 (48.2) | 260 (46.8) | 1155 (56) | 172 (10.1) | – |
| Mestizo/Hispanic | 701 (65.0) | 537 (44.2) | 185 (5) | – | 268 (15.6) | 78 (14.1) | – | – | – |
| Asian | – | – | – | (~80) | 271 (15.7) | – | – | 1497 (87.7) | – |
| Other | 11 (1.0) | 18 (1.5) | – | – | 71 (4.1) | – | 138 (7) | 38 (2.5) | – |
| **Age at diagnosis, years** | Median (IQR): 27 (20-35) | Mean (SD): 30 (12) | Mean (IQR): 33.1 (2.4-41.7) | – | – | – | Mean: 33 | Median (IQR): 29.0 (21.0–40.0) | – |
| **Age at cohort entry/registry enrollment, years** | Median (IQR): 35 (27-44) | – | Mean (IQR): 35.2 (24.2-43.9) | Median (IQR): 40 (31-51) | Mean (SD): 35.0 (13.4) | Mean (SD): 38.1 (11.4)-41.8 (13.9)^a^ | – | Median (IQR): 40.4 (31.2–50.6) | Median (range): 31 (17–58) |
| **Socioeconomic level,**  **n (%)** |  |  |  |  |  |  |  |  |  |
| Low-Medium | 438 (41.0) | 733 (61.3)^b^ | – | – | – | – | – | – | – |
| Medium-High | 629 (59.0) | 463 (38.7)^b^ | – | – | – | – | – | – | – |
| **Education, mean (SD), years** | 13.3 (11-16) | 9.2 (4.4)-10.4 (4.3)^b^ | – | – | – | 10.7 (3.7)-14.2 (3.1)^a^ | – | – | – |
| **Current smoker, n (%)** | 54 (5.1) | – | – | – | 263 (15.3) | – | – | 82 (5.4) | – |
| **SLEDAI** | Median (IQR): 5 (2.0-11.0)^c^ | Mean (SD): 13.1 (8.3)^b,d^ | – | Median (IQR): 3.3 (1.5-5.3)^e^ | Mean (SD): 5.3 (5.3) | – | – | – | – |
| **SDI ≥1, n (%)** | 405 (37.7) | – | – | – | 130  (19.4) | – | – | – | – |
| **LN, n (%)** | 653 (60.3) | (51.7)^f^ | (29-44)^a^ | 692 (39.9) | 467 (27.1)^g^ | – | – | 851 (49.9)^h^ | 110 (100) |
| **Treatment, n (%)** |  |  |  |  |  |  |  |  |  |
| Corticosteroids | 512 (78.9) | (91.8)^b^ | – | (>80) | 1199 (69.6) | – | – | 1405 (82.3)^i^ | – |
| Antimalarials | 561 (86.2) | (74.7)^b^ | – | – | 1153 (67) | – | – | 1217 (71.3)^i^ | – |
| Immunosuppressants | – | (46.9)^b^ | – | – | 684 (39.7) | – | – | 1193 (69.9)^i^ | – |
| **LN associated variables, n (%)** |  |  |  |  |  |  |  |  |  |
| Proteinuria | 394 (60.8) | (4.5)^b,j^ | (30-44)^a^ | 567 (32.7) | – | – | – | – | – |
| Cellular casts | 121 (20.5) |  | (21-28)^a^ | 72 (4.2) | – | – | – | – | – |
| Hematuria | 230 (37.5) | – | (3-6)^a^ | 350 (20.2) | – | – | – | – | – |
| Pyuria | 147 (24.3) | – | (2-5)^a^ | 277 (16.0) | – | – | – | – | – |

GLADEL, Grupo Latinoamericano de Estudio del Lupus; LN, lupus nephritis; RELESSER, Spanish Society of Rheumatology Systemic Lupus Erythematosus register; SDI, Systemic Lupus International Collaborating Clinics Damage Index; SLEDAI, Systemic Lupus Erythematosus Disease Activity Index; SLICC, Systemic Lupus International Collaborating Clinics.

^a^Range across ethnic groups.

^b^Data were calculated as percentages from White, Mestizo, and African-Latin American ethnic groups (N=1196). ^c^SLEDAI-2K at cohort entry.

^d^Average of the maximum score for each patient. ^e^SLEDAI-2K time adjusted mean.

^f^Any renal manifestation.

^g^Active renal disease at baseline.

^h^Renal disease at baseline.

^i^Reported during follow-up.

^j^Combined proteinuria and cell cast metric.

**Supplemental Figure 1: Centers Participating in the GLADEL 2.0 Cohort**


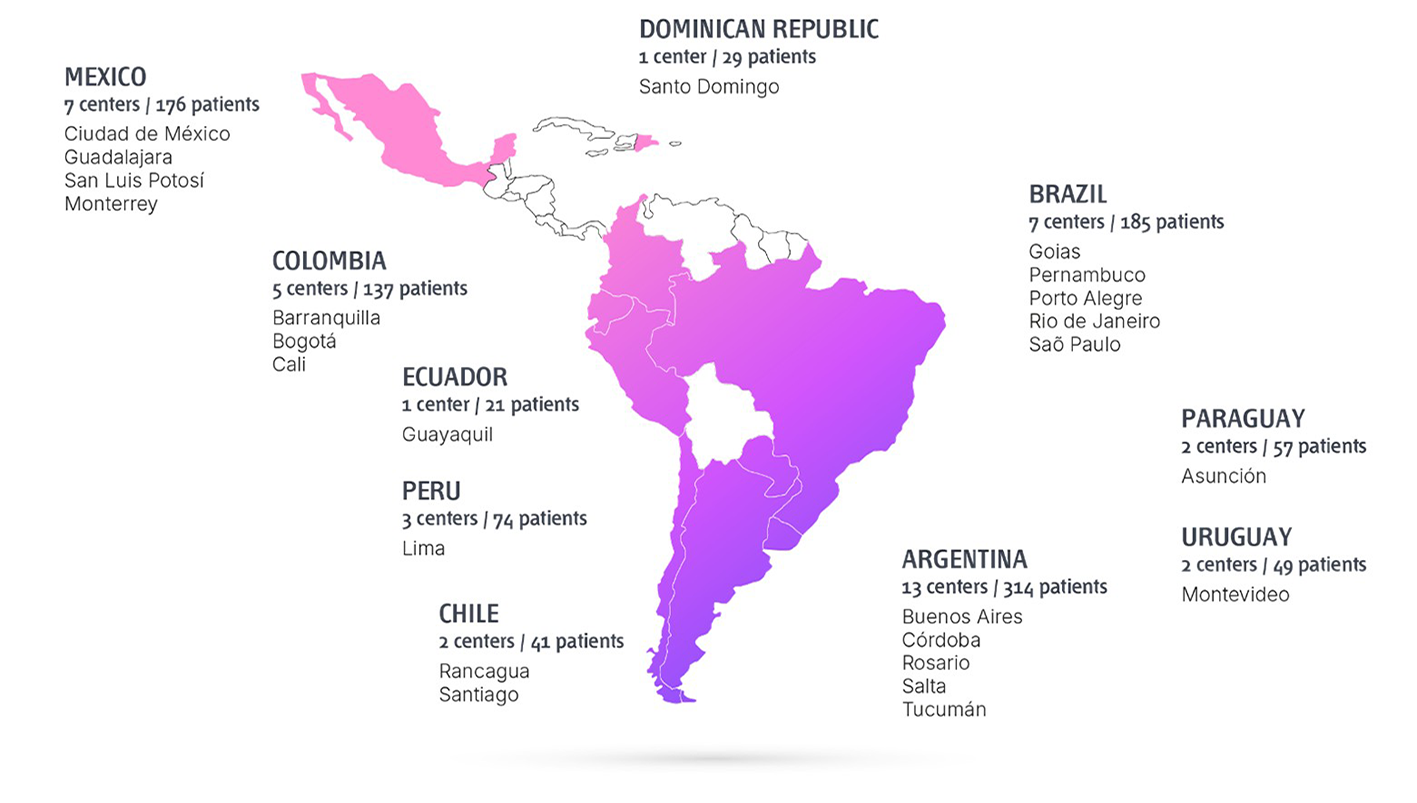


**Supplemental Figure 2: Cumulative Damage in SLE Groups at Cohort Entry**

***
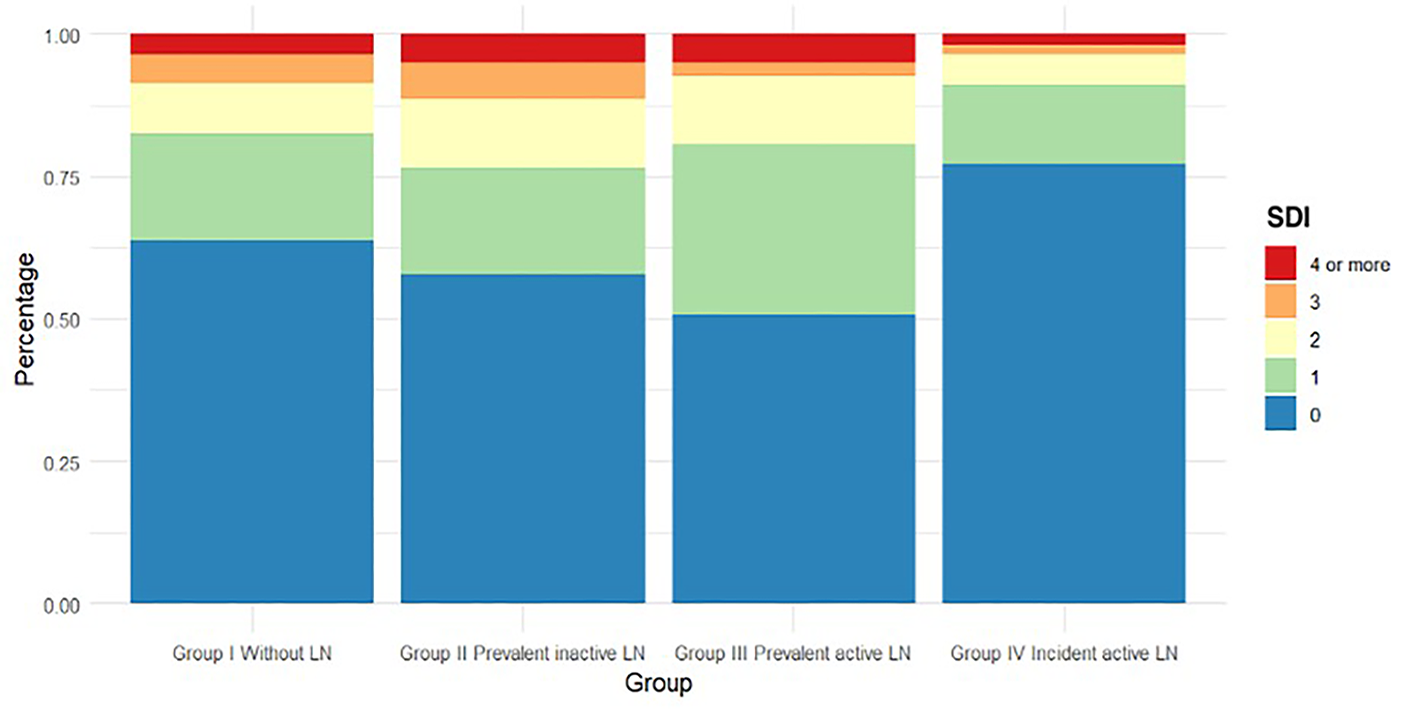
***

LN, lupus nephritis; SDI, Systemic Lupus International Collaborating Clinics/American College of Rheumatology Damage Index; SLE, systemic lupus erythematosus.

Group I: SLE, without renal involvement; Group II: SLE, with prevalent renal involvement, currently inactive; Group III: SLE, with prevalent renal involvement, currently active; Group IV: SLE with incident renal involvement.
